# Wireless Underground Sensor Communication Using Acoustic Technology

**DOI:** 10.3390/s24103113

**Published:** 2024-05-14

**Authors:** Md Adnan Al Moshi, Marcus Hardie, Tanveer Choudhury, Joarder Kamruzzaman

**Affiliations:** 1 Centre for Smart Analytics (CSA), Federation University Australia, Churchill, VIC 3842, Australia; t.choudhury@federation.edu.au (T.C.); joarder.kamruzzaman@federation.edu.au (J.K.); 2 Cooperative Research Centre for High Performance Soils (Soil CRC), Callaghan, NSW 2308, Australia; marcus.hardie@utas.edu.au; 3 Tasmanian Institute of Agriculture (TIA), University of Tasmania, Sandy Bay, Hobart, TAS 7005, Australia

**Keywords:** smart cities, wireless underground sensor network (WUSN), IoT-based moisture sensor, acoustic communication, below ground communication, sustainable agriculture

## Abstract

The rapid advancement toward smart cities has accelerated the adoption of various Internet of Things (IoT) devices for underground applications, including agriculture, which aims to enhance sustainability by reducing the use of vital resources such as water and maximizing production. On-farm IoT devices with above-ground wireless nodes are vulnerable to damage and data loss due to heavy machinery movement, animal grazing, and pests. To mitigate these risks, wireless Underground Sensor Networks (WUSNs) are proposed, where devices are buried underground. However, implementing WUSNs faces challenges due to soil heterogeneity and the need for low-power, small-size, and long-range communication technology. While existing radio frequency (RF)-based solutions are impeded by substantial signal attenuation and low coverage, acoustic wave-based WUSNs have the potential to overcome these impediments. This paper is the first attempt to review acoustic propagation models to discern a suitable model for the advancement of acoustic WUSNs tailored to the agricultural context. Our findings indicate the Kelvin–Voigt model as a suitable framework for estimating signal attenuation, which has been verified through alignment with documented outcomes from experimental studies conducted in agricultural settings. By leveraging data from various soil types, this research underscores the feasibility of acoustic signal-based WUSNs.

## 1. Introduction

Over the last decade, precision agriculture (PA) techniques [1,2] have emerged as potential solutions for increasing agricultural productivity to meet the demands of a growing global population and to adapt to future climate change. The integration of Internet of Things (IoT) devices [3,4,5] into agriculture has revolutionised farming practices, enabling farmers to make data-driven decisions for optimizing resource utilisation and enhancing crop yield. Among the most commonly used IoT devices in agriculture are soil moisture sensors, which provide real-time data on soil moisture levels and are instrumental in guiding irrigation scheduling [6,7,8]. As the world increasingly moves toward smart cities, the adoption of precision agriculture technologies has become paramount. These technologies contribute to efficient resource management, reduce environmental impact, align with broader initiatives for building smart cities, and promote environmental sustainability. The global agriculture sensor market has been growing steadily and is predicted to reach USD 2.56 billion by 2026, exhibiting a compound annual growth rate (CAGR) of 11.04% [9], while the market for soil moisture sensors alone is expected to grow over 13.7% over the same period [10]. This growth reflects the increasing recognition of the importance of PA agriculture in ensuring food security and environmental sustainability in the face of global challenges.

**Figure 1:**
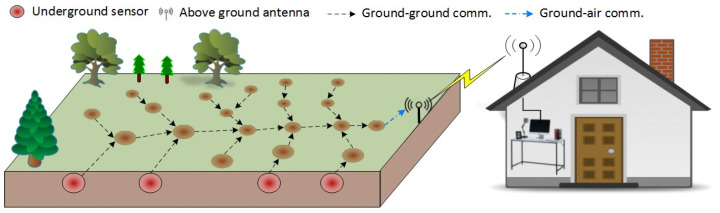
Underground-to-underground multi-hop communication in a farm setting with the last buried node communicating through the soil and air to an above-ground gateway.

Current IoT-based soil moisture monitoring systems typically consist of a power supply (often a solar panel), a data port, wireless telemetry nodes, and antennas for communication with remote devices. Being above ground, this setup poses several challenges, including risks of damage from heavy farming machinery, livestock, pests, flooding, and bushfires. One solution to mitigate these above-ground hazards is to place all components underground and establish a wireless communication system using below-ground-to-below-ground (BG2BG) nodes to transmit data from sensors out of the crop growing areas up to the cloud and the end user. In Figure 1, communication nodes and sensors are strategically placed underground throughout the paddock for full coverage, communicating wirelessly with one other. The last node is connected to an above-ground antenna, which transmits data to end devices.

For a wireless underground communication system to be suitable in an agricultural context, the transducers need to be small enough to fit into a borehole having a radius of around 10 cm [11], and they can be buried at variable depths [12] without restricting root growth. These transducers should be capable of sending data from BG2BG devices in any soil type and under variable moisture conditions. The received signal strength should be −100 dBm or a minimum of 4.5 Hz for geophone detection [13]. The system should be able to transmit sensor data from 1 to 10 times a day [14].

Recent underground communication technology based on radio frequency (RF) can only send data within the range of 4–20 m, which is inadequate for implementing most precision agriculture applications requiring transmission ranges between 50 to 100 m [15]. The low transmission range of BG2BG communication using existing RF/EM-based systems is due to the high degree of signal attenuation in soil. Therefore, concerns regarding coverage and power-efficient wireless underground technology persist. In this context, acoustic waves hold considerable potential to outperform RF signals in their ability to propagate data through the soil, primarily due to acoustic transducers using lower frequencies, resulting in lower attenuation and dispersion [11,16]. To evaluate the potential performance of acoustic BG2BG communication networks, a theoretical understanding of how soil properties influence wave propagation and attenuation is required. While models based on RF wave propagation exist for exploring the impacts of soil texture (clay content), moisture or volumetric water content (VWC), electrical conductivity, and density, similar models for acoustic wave propagation through soil are currently missing.

The authors are aware that there is currently no research exploring the acoustic propagation characteristics and understanding the influence of transmission frequency, moisture, and compaction (bulk density) on signal attenuation and thereby attainable transmission range in soil for agricultural applications. In addressing these knowledge gaps, this study makes the following contributions to developing a BG2BG acoustic communication network for agricultural soils:

(i) Investigation of existing literature for acoustic wave propagation models in soil for agricultural application. This work presents a comparative summary based on some key parameters of soil which would allow us to discern a suitable theoretical framework capable of analysing acoustic signal attenuation below ground.
(ii) The analysis of existing acoustic models for their suitability for agricultural soil. This is the first time such analysis is done from an agricultural usage perspective.
(iii) The analysis of acoustic signal attenuation involves the consideration of pivotal agricultural soil parameters such as soil composition, compaction, and moisture level which impact the attenuation of acoustic waves underground. The findings of this study will guide the development of a BG2BG wireless communication system with a better transmission range compared to existing technology including RF.

The paper is structured as follows: Section 2 reviews the existing wireless underground sensor network (WUSN) technologies and explores the potential of WUSNs based on acoustic technology. Section 3 provides a review of theories related to underground acoustic propagation to identify a model capable of simulating acoustic attenuation under diverse conditions in agricultural soil. Section 4 elucidates the behaviour of acoustic signals through the soil using the selected model and real-world soil data. Finally, Section 5 presents the study’s conclusion.

## 2. Modern WUSN Technologies and Advancement of Acoustics

In the last five decades, numerous attempts to establish underground wireless communication using Electromagnetic (EM) waves have been undertaken. In one experiment, the authors achieved communication between boreholes at a rate of 10 bps using repeaters [17]. EM-based systems have very low coverage (a few meters) due to severe path loss in soil [18]. The solution was to use low operating frequency for EM waves that have a better penetration capacity compared to high-frequency waves [19]. However, moisture content has a severe impact on the EM signal, and low-frequency transmission requires a larger antenna to receive a low-frequency signal (which is impractical to install in a small borehole) [20].

A multi-hop magnetic induction (MI) based system was investigated due to its robust nature [21]. However, the challenge of improving transmission range persisted due to the high attenuation of magnetic field over distance. Besides, perfect orientation (point-to-point) between the transmitter and receiver is required to achieve a better signal reception which is hard to maintain in an underground environment. More importantly, the MI-based system attenuates differently in different layers of the soil [22].

The mud pulse telemetry (MPT) system was introduced to monitor oil and gas [23]. In MPT, mud circulation was used to cool down the drill, convey information, and balance the pressure. The data rate for the MPT system was very low (1 bps), and the signal is attenuated due to the mud type, joints in the drill string, signal frequency, the diameter of the string, and borehole depth [24].

Research has shown the potential of a multi-hop radio frequency (RF) based communication network for reliable data transmission [15]. A 433 MHz LoRa radio technology at +23 dBm transmission power in four in situ soils was used for wireless underground sensor networks in an agricultural paddock. This research revealed that factors like burial depth, antenna type, transmission power, receiver height above the ground, and soil type have significant impacts on signal transmission. They deployed the system that could receive signal BG2BG only in the range of 4 to 20 m. In addition, low-frequency RF and EM signals require large antennae to generate and detect signals [25], which is logistically challenging to install in soil environments. Additionally, soil permittivity varies with the change in moisture content. As a result, the resonant frequency and bandwidth of the antenna change due to the variations in the return loss characteristics [26].

Geologists have used acoustic waves to search for oil, gas, and buried artefacts, monitor earthquakes, and identify leaks in underground pipes [27] and as smart drilling for reservoirs [28]. In these applications, acoustic methods are categorised by how signals are generated, either active or passive. In the active method, an artificial explosion or vibration is used to generate a signal, which is then used to estimate soil or rock properties. For example, in [11] the harmonic sound was produced by an above-ground thumper which was transmitted to an underground sensor to operate a drill located at 100 m depth. In contrast, passive waves are generated mainly by natural disasters such as earthquakes and volcanic eruptions. Underground sensors are placed in the proximity of the passive wave generation area that detects the infrasonic signals used to predict natural disasters. However, there is inadequate research conducted for below-ground sensor communication within agricultural contexts across diverse soil compositions and parameters using acoustic wave-based technology, thus presenting an opportunity for this study to make a meaningful contribution.

Most BG2BG acoustic transceivers use lower frequency wavelengths (40 Hz–100 Hz), which cause lower attenuation and dispersion, resulting in better signal strength and longer detection distances. In theory, acoustic technology has the potential to outperform RF and EM-based BG2BG technologies through increased transmission distance, reduced signal attenuation, and lower power consumption. Acoustic transducers such as voice coil actuators and geophones do not require an antenna to send or receive signals. Additionally, the acoustic wave produced by acoustic transducers has a better impedance matched with soil, ensuring low spatial variability [11].

Acoustic transducers are low-powered, and power-efficient modulation techniques (on-off keyed modulation) are used. Hence, the acoustic approach can reduce power consumption and has the potential to greatly enhance battery life to 45 months [11] compared to several months for the RF-operated system. 

A comparative study of different WUSN technology has been presented in Table 1. This comparative study demonstrates the prospect of using acoustic technology to meet the requirement of a feasible wireless communication system for agricultural applications.

## 3. Acoustic Wave Propagation Model through Soil

Biot’s [29] theory characterises acoustic wave propagation through porous media including soil. The theory states the existence of two primary waves (Type I and Type II) and one shear wave when sound propagates through porous media. It also states the particle motion is in phase with the primary waves, whereas in the shear wave, the particle motion is vertical to the motion of the fluid. Researchers have observed that Biot’s shear wave occurred in the liquid-saturated porous media [30] in homogeneous, isotropic soil using an impulse source close to the surface. They found that the ‘shear wave’ and another surface wave (‘Rayleigh waves’) are formed when the acoustic wave reaches the surface of the ground.

Although Biot’s theory has been useful for understanding the properties of acoustic waves in homogeneous and isotropic media, however, soil is spatially heterogeneous, anisotropic [31], and variably saturated. As Biot’s theory is only applicable to a saturated porous medium, it is not an ideal model for agricultural soils in which soil moisture tends to range between field capacity (moisture after drainage) and the permanent wilting point at which most plants will die.

The Brandt [32] model considered soil as a spherical model where different particles are stacked randomly. The theory assumed that the pore space could only be occupied by a single type of fluid, precluding the coexistence of both gas and liquid. It explained the impact of pressure, porosity, and liquid on the speed of sound, assuming that fluid and solid particles move together with the same displacement when stress is applied. This assumption limits the idea of elastic wave propagation because when an elastic wave propagates energy dissipates due to the relative motion of fluid with respect to solid aggregates. Moreover, the fluid and gas can present together in soil [33].

Brutsaert’s [34] theory explains acoustic pulse transmission for granular and unconsolidated porous mediums. According to this theory, acoustic sources generate three compression waves and one shear wave. This model is known as a velocity model which yields the velocity of the elastic wave for which it is necessary to know the interstitial effects, total density, and the effective pressure of the soil specimen which is related to the degree of saturation [35,36]. The measurable parameter in this model is the signal velocity, not the signal strength, hence, it is not suitable for modelling attenuation. Furthermore, this model does not include a wave equation which is necessary to determine the detectable signal strength. Also, this model requires the transceivers to be placed 30–70 cm apart, which makes it impractical for the BG2BG network as the number of transceivers will be enormously high to cover farming land.

Unlike the above models and the models listed in Table 2, the Kelvin–Voigt model (Section Kelvin–Voigt Model) characterises soil channels as viscoelastic media [37]. The Kelvin–Voigt model can calculate the attenuation due to both material and geometric damping, which are together responsible for the total acoustic signal path loss. The model provides a complete wave equation enabling the exploration of acoustic wave propagation in soil. Even though the Kelvin–Voigt model has been used by geologists and geotechnical engineers to estimate earthquake waves, no research has been undertaken to study the use of this model for underground sensor communication in agricultural soils. The characteristics of the selected acoustic models are presented in Table 2 in which the Kelvin–Voigt model demonstrates potential for modelling signal propagation in agricultural soils.

### Kelvin–Voigt Model

**Figure 2:**
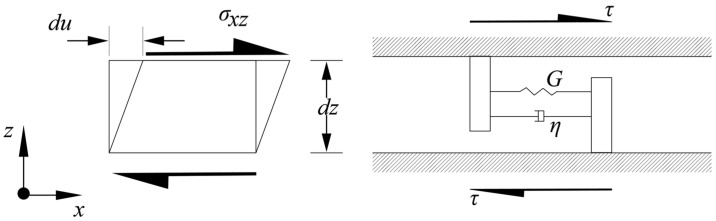
Horizontal shear applied to a thin Kelvin–Voigt solid. Total resistance to shearing deformation is the sum of an elastic (spring) component and a viscous (dashpot) component [41].

Kramer [41] proposed a wave propagation model that considers the attenuation due to material damping. To articulate the viscoelastic behaviour, the soil is considered a Kelvin–Voigt solid, which has both viscous and elastic properties (Figure 2). The solids have a parallel arrangement of linear springs and a dashpot. In general, the shear stress–strain relationship for the Kelvin–Voigt model can be expressed as the sum of an elastic part (proportional to strain) and a viscous part (proportional to strain rate), as in Equation (1).

(1) $$\tau =G\gamma +\eta \frac{\partial \gamma }{\partial t}$$

where *τ* is the shear stress (*τ* = *σ_xz_*), *γ* is the shear strain (*γ* = $\frac{\partial u}{\partial z}$), and *G* is the shear modulus and *η* is the viscosity of the material. The acoustic wave equation in the direction is given in Equation (2), which can be derived from Equation (1).

(2) $$u(z,t)=Ae^{k_{2^{z}}}e^{i\left(\omega t-k_{1}z\right)}$$

where *u*(*z*) is the displacement of the wave in time *t*, and $Ae^{k_{2^{z}}}$ denotes the amplitude attenuation of the wave due to damping, where *A* depends on the distance between the source and receiver, and *k*_1_ and *k*_2_ are damping coefficients (Equation (3)). *z* is the distance, *ω* is the angular velocity which depends on the frequency *f*, and *ω* = 2*πf*:

(3) $$k_{2}^{2}=\frac{\rho \omega^{2}}{2G\left(1+4\zeta^{2}\right)}\left(\sqrt{1+4\zeta^{2}}-1\right)$$

In Equation (3), only the negative root of *k*_2_ has physical significance as it depicts the reduction of signal amplitude due to attenuation. As a result, from Equation (3), it is seen that the body wave amplitude $Ae^{k_{2^{z}}}$ decreases exponentially with distance *z*. *k*_2_ depends on soil parameters, namely, bulk density (*ρ*), damping ratio (*ζ*), shear modulus (*G*), and viscosity (*η*). Bulk density (*ρ*) can be calculated using the porosity parameter of the soil given by Equation (4) [42] in which 2.65 (g/cm) is the particle density of quartz.

*ρ* = (1 *−* porosity of the medium) × 2.65 (4)

The damping ratio (*ζ*) measures the energy dissipation of the acoustic source through the soil and can be calculated by Equation (5) if the source frequency (*f*), viscosity (*η*), and shear modulus (*G*) are known.

(5) $$\zeta =\frac{\eta \omega }{2G}$$

The elastic energy decreases when it is distributed over a greater volume of the material. This phenomenon is called radiation damping or geometric damping. The far-field amplitude attenuation, due to geometric damping, is $\frac{1}{z^{2}}$ for the primary waves (Type I and Type II) [43]. The total path loss (*L_PL_*) due to attenuation has two components: (i) geometric damping (*L_GD_* = $\frac{1}{z^{2}}$), and (ii) material damping (*L_MD_* = $e^{k_{2^{z}}}$). The *L_PL_* is presented in Equation (6) in dB.

*L_PL_* = 10 log_10_*L_GD_* + 10 log_10_ *L_MD_* = −20 log_10_*z* − 4.34*k*_2_*z* (6)

## 4. Methods

### 4.1. Software

We investigated the behaviour of an acoustic signal and its changes with respect to different soil properties, namely, moisture (VWC) and compaction (bulk density) using Equations (3), (5), and (6), derived from the Kelvin–Voigt model. The key soil parameters employed for simulation purposes included bulk density, viscosity, and shear modulus, alongside the frequency of the acoustic source and the separation distance between the transmitter and receiver. Simulations were conducted in MATLAB R2020a using the data for four types of soil, namely, clay, silty clay loam, clay loam, and sandy loam which represent key soil texture groups used in agriculture.

### 4.2. Soil Properties

For the simulation purpose, knowledge of bulk density (*ρ*), viscosity (*η*), and shear modulus (*G*) data of soil is required. Soil bulk density data can be calculated from the porosity (Equation (5)). Viscosity data can be measured using a viscometer or estimated from the Atterberg limit data for different texture groups [44]. We used secondary sources [45,46] to prepare Table 3 for the simulations.

### 4.3. Data Analysis

Three experiments were conducted in MATLAB. Experiment 1 explored the effect of frequency on signal strength degradation and the distance it can travel before attenuating below an assumed value of −100 dBm threshold, beyond which the signal was no longer detectable. For example, in wireless communication systems if the distance increases ten times, then path loss increases by 20 dB and the detectable received signal power threshold level reduces from −80 dBm to −104 dBm [47]. Since very little research has been conducted in underground acoustic communication, we assume the threshold at −100 dBm. The influence of frequency on signal quality was investigated by altering frequency between 20–100 Hz in 10 Hz increments whilst other soil parameters (Table 3) were constant. This frequency range was chosen because a 100 bps data rate is common for underground applications for which 100 Hz can provide sufficient bandwidth [11].

Experiment 2 examined the effect of compaction or bulk density (*ρ*) on signal degradation and the transmission distance covered using 40 Hz frequency in clay loam soil at two contrasting moisture contents using the data [48] presented in Table 4.

Experiment 3 investigated the effect of different moisture contents (VWCs) in clay soil on acoustic signal degradation with distance, at an operating frequency of 40 Hz. A frequency of below 40 Hz does not have sufficient bandwidth to send the soil data. For this experiment, secondary data [44,49] for viscosity and shear modulus have been used at a range of moisture levels (5%, 18%, 25%, 40%, 48%, and bulk density of 1.39 g/cm^3^). For moisture exceeding 48%, the clay soil reaches field capacity.

## 5. Results

### 5.1. Impact of Frequency on Acoustic Signal Propagation

**Figure 3:**
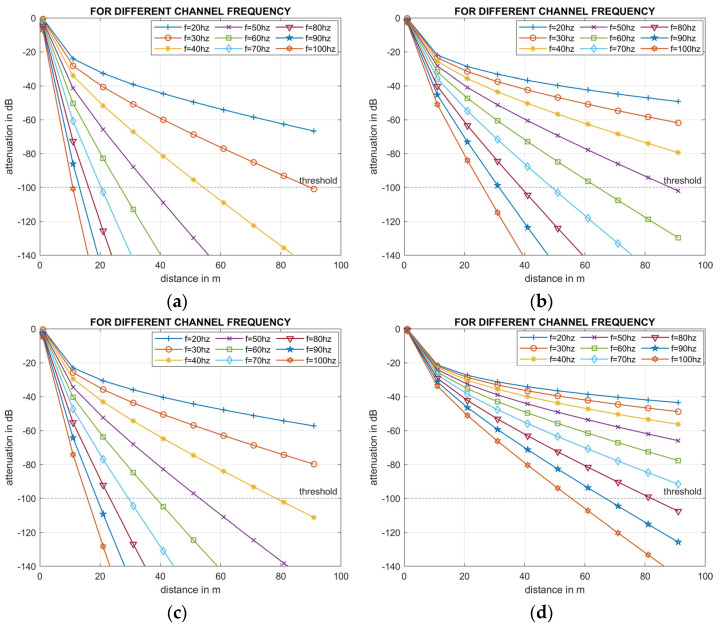
Impact of frequency on signal propagation for various soil types: (**a**) clay, (**b**) silty clay loam, (**c**) clay loam, and (**d**) sandy loam at fixed soil moisture.

Attenuation increased with frequency for all four soil types (Figure 3). These figures help determine the optimal frequency range for data transmission. Results indicate that to achieve a transmission distance of 55 m and an attenuation level greater than −100 dB, the signal frequency would need to range from 40 Hz in clay to 100 Hz in sandy loam. The simulation results also show that as the clay content decreased the transmission distance increased. Hence, in sandy loam with only 20–30% clay, a transmission distance of over 90 m can be achieved at a frequency between 10 Hz to 70 Hz.

### 5.2. Impact of Compaction or Bulk Density on Signal Propagation

**Figure 4:**
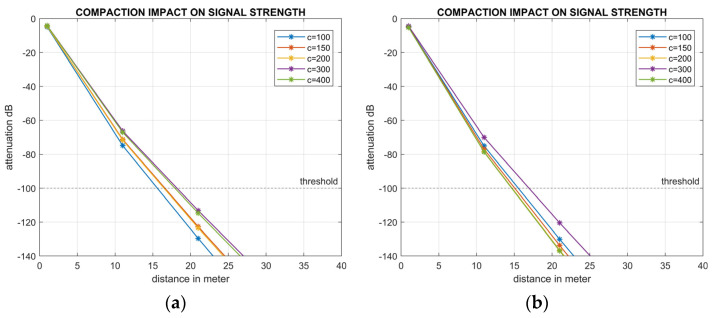
Impact of compaction at different moisture levels: (**a**) 10% moisture level, and (**b**) 17% moisture level on signal propagation.

Simulation results demonstrate that the transmission distance increased from 15 m to 18 m in clay loam soil at 10% moisture when compaction increased from 100 kPa to 400 kPa (Figure 4a). At 17% moisture, the transmission distance was 18 m at 300 kPa, which was reduced to 15 m at 400 kPa (Figure 4b).

### 5.3. Impact of Moisture on Acoustic Signal Propagation

**Figure 5:**
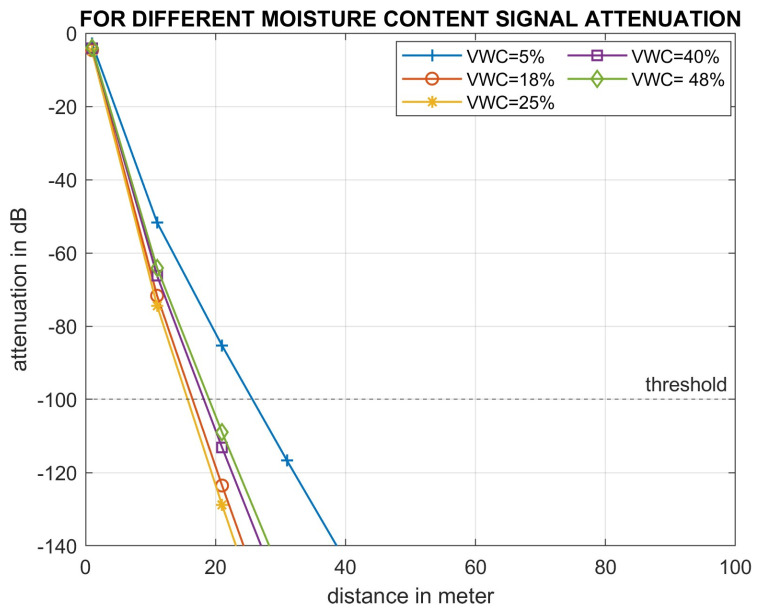
Impact of moisture content (% vol) on signal propagation.

Results show that in clay soil at a bulk density of 1.3 gm/cm^3^ and a 40 Hz frequency, an acoustic signal of above −100 dB can be detected at a distance of 25 m when the soil moisture (VWC) is 5% (Figure 5). The transmission distance was reduced to 17 m when the moisture level increased to 25%.

### 5.4. Model Verification and Comparison of Results

The model simulation results have been compared with known experimental results reported in studies in literature. Though these studies were not conducted using agricultural soils they can provide a basis for comparison to verify our model.

Yang et al. [11] tested a compact underground acoustic system. In their experiments in field trials, acoustic transducers and receivers were installed in boreholes of 20 cm in diameter at depths ranging between 30 cm and 150 cm in natural soil conditions typically encountered in construction or agricultural sites. Measurements were conducted by maintaining the transducer and receiver distances 15 m to 50 m distances from each other. Using the frequency ranging from 20 Hz to 80 Hz, they reported attenuation ranging from −70 dB to −105 dB. Our simulation also showed a similar trend in signal attenuation. For example, in the case of sandy loam soil (Figure 3d), at 60 Hz, signal attenuation ranged from −40 dB to −110 dB. They also found that reliable communication at a 2 bps data rate was achievable at a range of up to 50 m in the outdoor field experiment which matches with results from our simulation which shows a detectable signal >−100 dB in the range of 15 m to 90 m for clay and 60 m to more than 90 m for sandy loam. In [50], Pal et al. summarised laboratory and field experiments for different types of soils over a wide range of frequencies. Their study indicated that silty clay experienced significantly higher attenuation compared to sandy loam soil, a finding that was also supported by our simulation results.

The comparison with results from various sources reinforces the model’s validity and highlights its ability to be used for a range of soils or porous media. The overall findings underscore the reliability of the Kelvin–Voigt model and its usefulness as a predictive tool to analyse the performance of acoustics for underground sensor communication in agricultural soil.

## 6. Discussion

The findings from simulations suggest that acoustic transmission exhibits superior performance compared to RF transmission within the soil environment. Nevertheless, the transmission distance of acoustic transmission is significantly influenced by soil moisture levels and to a lesser extent by soil types. First, the simulations illustrated that a transmission distance of greater than 90 m is achievable for signal frequencies between 20–30 Hz. However, as the transmission frequency increased, signal attenuation caused a reduction in transmission distance. Consequently, a frequency of 40 Hz was chosen for acoustic transmission in the soil to accommodate a bandwidth sufficient for transmitting soil data at 100 bps. Although this frequency limits the transmission distance to 55 m. Notably, the change from sandy loam to clay soil resulted in a decrease in the acoustic signal’s transmission range from over 90 m to 55 m due to increased clay content. Oelze et al. [28] asserted that this increase in clay content leads to greater porosity, diminishing the interaction of the acoustic signal with the soil framework, resulting in the generation of a secondary wave (shear wave) that attenuates faster than the primary waves.

Second, the results indicate a positive correlation between increased compaction or bulk density (*ρ*) and the transmission distance of acoustic signals (Figure 4a). Consistent moisture levels across soil types combined with higher degrees of compaction promote solid-to-solid contact, subsequently elevating viscosity and reducing material damping, ultimately leading to decreased attenuation. Conversely, elevated moisture levels decrease viscosity due to increased solid-to-solid gaps, intensifying attenuation, and reducing transmission distance [28]. In Figure 4b the maximum transmission distance at 17% soil moisture is close to at 10% moisture. This happens due to an increase in cohesion among soil aggregates at 17% moisture. However, anomalies in transmission distance with increasing compaction, as observed in both Figure 4a,b, may be attributed to the stratified impact of moist soil layers [28]. The bulk density data presented in Table 3 for clay and clay loam soils represent values at the upper limits of agricultural soil. Typically, agricultural soil maintains a BD below 1.5 g/cm^3^ to facilitate optimal root growth for plants [51]. The third set of experiments demonstrates that alterations in BD resulting from changes in compaction minimally affect signal propagation, with transmission distance variations remaining within 5 m (Figure 4a,b). 

Consequently, it is reasonable to anticipate that in situ bulk density will exert minimal influence on signal propagation. However, further validation through laboratory testing is warranted to affirm this assertion.

Third, experimental observations highlighted the substantial impact of soil moisture on the degradation of acoustic signals. The plotted data (Figure 5) demonstrated that increased moisture content adversely affected signal propagation due to reduced viscosity and shear modulus [49,52]. Additionally, the plot illustrated that for moisture levels exceeding 40%, the rate of signal attenuation with increasing moisture is less pronounced compared to levels at 18% or 25% moisture. This reduction in attenuation is attributed to increased cohesion among soil aggregates as moisture surpasses the 40% threshold, enhancing particle-to-particle contact and thereby improving acoustic coupling [53].

## 7. Conclusions

Soil is a complex media, which has both viscous and elastic characteristics. Simulation results based on the Kelvin–Voigt propagation model are highly useful for exploring the effect of signal frequency, soil moisture, and bulk density on acoustic signal attenuation caused by geometric and material damping. Results show that attenuation increases with increasing transmission signal frequency, increasing clay content, soil moisture, and compaction. To develop a BG2BG communication system using acoustic waves for agricultural soil in farmland, this study indicates that, under practical considerations, a transmission distance of about 55 m in moderately compacted clay soil and 90 m in sandy loam soil is achievable depending on the soil moisture content. The model and simulation results demonstrate the potential benefits of using acoustic technology for the BG2BG network compared to RF and EM technologies.

Even though the simulation and theoretical investigation of this study constituted the foundation in guiding the design of an acoustic underground communication system, one limitation involves the potential enhancement of its validity through rigorous validation by laboratory and field trials. Future investigations will centre on conducting both laboratory and field trials. The laboratory trials will involve the preparation of a wooden container using agricultural soil to study the attenuation effects, moisture influence, and data transmission rates pertinent to acoustic technology. This experimental setup will entail the utilisation of a prototype voice coil actuator (VCA) as the transmitter, soil sensors, and a geophone; all interconnected with a Raspberry Pi. Similarly, the prototype will be deployed within agricultural fields to evaluate the viability and applicability of acoustic wave-based technology for below-ground data transmission.

## Figures and Tables

**Table 1:** Comparative study of various technologies for potential use in the below-ground communication network, particularly focusing on their suitability in the agricultural context.

WUSN Technologies	Key Parameters			Comment
	Coverage	Attenuation	Data Rate	
EM	Few meters	High	10 bps	Mostly used for seismic exploration and down-hole monitoring. Low coverage. Lack of low-frequency antenna.
RF	5–20 m	High	Tens of bps	Used in agriculture. High path loss due to increase in frequency and moisture content.
MI	Tens of meters	Low	In kbps	Used for down-hole telemetry. Low coverage. Maintaining the perfect orientation of the antenna is impractical.
MPT	Thousands of meters	Medium	10–20 bps	High data rate. Complex system. Mostly used for down-hole telemetry.
Acoustic	Inadequate information in the agricultural context; requires further study.	Requires further study	Tens of bps	Good transmission range. No antenna is required, transducers can be placed in the borehole. Inadequate application in an agricultural context.

**Table 2:** Comparative summary of acoustic wave propagation models through the soil.

Model Name	Channel Characteristics Considered				Comment
	Anisotropic	Attenuation Effect	Viscous	Elastic	
Biot’s Theory [29]	No	NM *	No	Yes	Based on the assumption of small strains and is valid for low-frequency acoustic wave propagation.
Brandt’s Model [32]	Yes	NM *	NM *	NM *	Relative motion between solid and fluid has not been considered.
Brutsaert’s Theory [34]	No	No	NM *	NM *	The primary focus is on one-dimensional flow profiles and does not adequately address spatial variations.
Gassmann’s Model [38]	No	No	No	Yes	Porosity remains unchanged with different saturating fluids which is not the case in agricultural soil.
Elastic wave propagation Model [39]	No	No	No	Yes	The primary application is the measurement of rock rather than agriculture.
Ray Tracing Method [40]	No	No	NM *	Yes	Suitable for high-frequency seismic waves due to the dependence on the idea of narrow ray bundles.
Kelvin–Voigt Model [37]	Yes	Yes	Yes	Yes	The wave equation estimates the attenuation of acoustic waves and incorporates the influence of soil parameters of agricultural soil.

* NM = Not Mentioned.

**Table 3:** Soil parameter values were used for the Kelvin–Voigt model.

Soil Texture Type	Clay Content (%)	Bulk Density (gm/cm^3^)	Viscosity, (Pas)	Shear Modulus, (MPa)
Clay	35–55	1.30	1019	2.4
Silty Clay Loam	25–40	1.41	1293	4.3
Clay Loam	25–35	1.40	1024	5.7
Sandy Loam	10–20	1.45	996	9.3

**Table 4:** Clay loam soil viscoplastic parameter.

Compaction Level (KPa)	After Compaction (gm/cm^3^)	Viscosity (Pas)	
		VWC = 10%	VWC = 17%
100	0.98	55,218	53,670
150	1.32	119,080	86,620
200	1.57	145,800	104,270
300	1.88	235,110	195,510
400	2.30	283,100	169,110

## Data Availability

Data are contained within the article.

## Abbreviations

The following abbreviations are used in this manuscript:

PA	precision agriculture
RF	radio frequency
EM	electromagnetic
BG2BG	below ground-to-below ground
VWC	volumetric water content
WUSNs	wireless underground sensor networks
BD	bulk density
